# Claudin18.2 as a Promising Therapeutic Target in Gastric Cancer

**DOI:** 10.3390/cells14161285

**Published:** 2025-08-19

**Authors:** Agata Poniewierska-Baran, Paulina Plewa, Zuzanna Żabicka, Andrzej Pawlik

**Affiliations:** 1 Institute of Biology, University of Szczecin, Felczaka 3c, 71-412 Szczecin, Poland; agata.poniewierska-baran@usz.edu.pl (A.P.-B.); 241473@stud.usz.edu.pl (Z.Ż.); 2 Department of Physiology, Pomeranian Medical University, 70-111 Szczecin, Poland; paulina.plewa@pum.edu.pl

**Keywords:** claudin18.2, gastric cancer, biomarker, therapy

## Abstract

Claudin-18.2 (CLDN18.2) is an isoform of a tight junction protein and has emerged as a promising therapeutic target in gastric cancer (GC). CLDN18.2 is responsible for gastric homeostasis and protects epithelial cells from low pH conditions. Interestingly, CLDN18.2 expression is strictly restricted to the stomach, making it an ideal tumor marker. This narrative review presents the characterization and role of claudin 18.2 (CLDN18.2) as a promising biomarker in GC and a target for clinical therapies, more specifically CLDN18.2-targeted drugs and therapies including mABs (e.g., Zolbetuximab, Osemitamab, ZL-1211), bsAB, and CAR-T cell-based immunotherapies. We also summarize numerous ongoing worldwide clinical trials that are evaluating CLDN18.2 as a target for GC treatment. What seems to be crucial is that preclinical and clinical data indicate their high efficacy and safety.

## 1. Introduction

According to GLOBOCAN data from 2020 [1], gastric cancer (GC) is one of the top five leading causes of cancer-related deaths worldwide. Most deaths from GC occur after the age of 50, and the increased risk of developing GC is closely correlated with age. Despite advances in diagnostics and systemic treatment, the prognosis is still poorer for patients diagnosed with advanced GC, especially those with multiple metastases. The choice of appropriate treatment is mainly related to the location of the tumor but also to the formation of metastases. Surgery constitutes the basic treatment plan, which ensures long-term survival. In the absence of a surgical recommendation, chemotherapy is performed, aimed at reducing the tumor or delaying its growth through the administration of cytotoxic drugs. It is often combined with radiotherapy [2]. There have been many scientific studies, clinical trials, and reviews on immunotherapy in GC, including projects focusing on immune checkpoint molecules (ICPMs) and immune checkpoint inhibitors [3] and other biomarkers in GC like HER2 [4], CEA [5], Ca19.9 [6], miRNA [7], etc. Despite the enormous progress in research and discovery of modern therapies based on new molecular targets, there is still a lack of universal strategies for GC patients, hence the continuous search for new and promising therapeutic targets. Recently, claudin 18.2 (CLDN18.2) has joined this select group of biomarkers in GC. Claudins belong to a multigene family of tetra span membrane proteins that constitute the main structural component of tight junctions [8,9]. They play a critical role in forming paracellular barriers and channels that determine the permeability properties of epithelial and endothelial cells. Below, in the following chapters and sub-chapters, we present the characteristics of the claudin 18.2 protein family and its expression in physiological conditions and in neoplastic disease, focusing especially on GC. We also collected data on therapeutic strategies and clinical trials currently or soon to be conducted with CLDN18.2 as a target in GC therapy.

## 2. Characteristics of Claudin-18.2

### 2.1. Claudins as a Tight Junction (TJ) Protein

Claudins are the most important structural and functional components of integral tight junction (TJ) proteins. Tight junctions, or zonula occludens, located in the upper part of the lateral cell membrane, border the apical surface [10]. They form a continuous barrier separating the apical from the basolateral domain and constitute one of the forms of cell–cell adhesion in epithelia and endothelia [9]. Their presence is essential for maintaining tissue integrity.

Tight junctions have two main functions in epithelial cells, contributing to compartmentalization: the “gate” function, which regulates paracellular permeability, and the “fence” function, which is performed at the subcellular level. The gate function controls the permeability of the cell membrane to ions, water, and macromolecules, while the fence function preserves epithelial cell polarity [8].

By transmission electron microscopy, the TJ is observed as a set of points where the outer leaflets of the membranes of adjacent cells appear to fuse, obstructing the passage through the paracellular pathway of electrodense markers [10]. While being observed by freeze–fracture microscopy, TJs can be seen to be composed of complex networks of strands, which can be extremely variable in terms of number and complexity depending on the cell type [9].

Tight junction proteins are functionally divided into three groups: transmembrane proteins, adaptor proteins, and signaling proteins [11]. The transmembrane proteins, directly involved in the formation and barrier function of TJs, include claudins (the main structural component), occludin, tricellulin, and junctional adhesion molecules (JAMs) [8]. Adaptor proteins such as ZO-1, MAGI, cingulin, and paracingulin connect transmembrane proteins to the actin cytoskeleton and to signaling proteins [10].

### 2.2. Claudin Protein Family

The claudin family in mammals consists of 27 genes, which show tissue-specific and differential expression patterns, with potential for alternative splicing [11]. Based on a phylogenetic analysis of full-length amino acid sequences, claudins are divided into classic and non-classic types. Classic claudins exhibit stronger sequence homology than non-classic claudins and include Claudins-1–10, -14, -15, -17, and -19. Non-classic claudins include Claudins-11, -12, -16, -18, and -20–27. Claudin-13 is not expressed in humans; its expression has been observed exclusively in mice. Functionally, claudins are also categorized into barrier-forming proteins (e.g., Claudins-1, -3, -5, -11, -14, -18) and channel-forming proteins, which give tight junctions selective ion permeability [8,9,11].

Most CLDN genes have low intron content, and some lack introns altogether. The result of this is that the genes are typically small, on the order of several kilobases. Several pairs of CLDN genes are very similar to each other in sequence and are located in close proximity in the human genome, suggesting their shared evolutionary origins [9].

Structurally, claudins are highly similar, containing four transmembrane domains, a short N-terminal cytoplasmic domain (~4–7 amino acids), a variable C-terminal cytoplasmic tail (~21–111 amino acids), one short intracellular loop (~20 amino acids), and two large extracellular loops: ECL1 (~50–60 amino acids) and ECL2 (~24 amino acids) [11,12].

Transmembrane domains 1 and 4 are highly conserved, while domains 2 and 3 are more variable. Within ECL1, a conserved motif “GLW-X(2)-C-X(7,9)-[STDENQH]-C” is present in mammals, with two cysteines capable of forming disulfide bonds to stabilize the loop structure. Claudin-10b and Claudin-15 are exceptions, containing NLW instead of GLW [12,13]. The extracellular loops, especially ECL1, determine claudin function. ECL1 is crucial for ion selectivity and tightness of the junction, while ECL2 plays a stabilizing role and may affect the spacing between adjacent cell membranes. The C-terminal tail includes a PDX-binding motif, allowing interaction with cytoskeletal proteins (e.g., ZO-1, ZO-2, ZO-3, MUPP1, PATJ, MAGUK). Additionally, the carboxy-terminal tail upstream of the PDZ motif is necessary for directing the protein to the TJ complex and is essential for its stability and function [9]. This region is also a site for post-translational modifications, such as serine/threonine and tyrosine phosphorylation and palmitoylation, which can significantly influence claudin localization and function [9].

The expression of various isoforms, including alternatively spliced ones, allows cells to finely regulate barrier function. This can also affect immune responses and adaptation to environmental changes.

## 3. Claudin-18.2 and Its Physiological Expression

The CLDN18 gene, located on chromosome 3q22 [14], encodes Claudin-18.2, a member of the integral components of tight junctions. This gene spans approximately 35 kB, includes six exons and five introns, and its first exon exists in two alternative forms: 1a and 1b. This enables alternative splicing via two independent promotors, resulting in two isoforms: Claudin-18.1 and Claudin-18.2 [15]. These isoforms share 91% sequence identity, differing mainly in the first 69 amino acids on the N-terminus encoded by exon 1, which also affects the structure of the ECL1 loop. This amino acid difference can prevent the epitope of the CLDN18.2 protein from immunologically cross-reacting with the CLDN18.1 protein [15].

Promoter P1a contains a binding site for the transcription factor T/EBP (NKX2.1), whose activation drives Claudin-18.1 expression in alveolar lung cells [16]. Promoter P1b, specific to Claudin-18.2, contains a binding site for CREB (cAMP response element-binding protein). In other cell types, methylation of promoter P1b suppresses CLDN-18.2 expression, contributing to its highly specific expression pattern [15].

**Figure 1:**
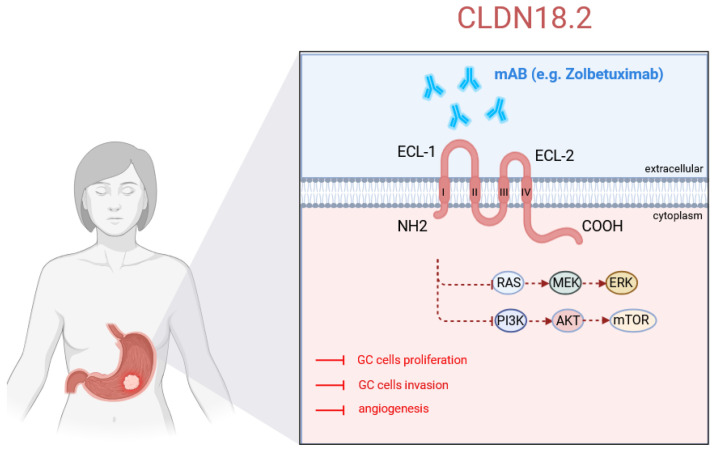
CLDN18.2 structure, expression, and mechanism of action in GC cells. Created in BioRender. Physiology, D. (2025) https://BioRender.com/4rpyait.

Structurally, Claudin-18.2 is similar to other claudins, including four hydrophobic transmembrane domains, two extracellular loops (ECL1: 73 amino acids; ECL2: 23 amino acids), a cytoplasmic loop, and both N- and C-termini located inside the cytoplasm. In ECL1, the conserved motif [GN]-L-W-x(2)-C-x(7,9)-[STDENQH]-C contains two cysteines (positions 52 and 63) capable of forming a disulfide bridge, stabilizing the loop’s structure. Additionally, cysteines at positions 103 (cytoplasmic loop) and 193 (C-terminal) allow for palmitoylation, a lipid modification crucial for membrane localization and protein function. The C-terminal cytoplasmic domain (71 amino acids) contains a PDZ-binding motif, facilitating interactions with adaptor proteins of tight junctions. These interactions are essential for maintaining tight junction integrity and their connection to the actin cytoskeleton (Figure 1) [17].

Claudin-18.2 exhibits highly selective expression, restricted to differentiated gastric parietal cells, and is not present in progenitor or undifferentiated cells within the gastric mucosa stem cell niche [14]. It plays a crucial role in maintaining epithelial barrier continuity, cell polarity, and selective permeability to Na^+^ and H^+^ ions in the acidic environment of gastric acid [15,18]. Claudin-18.2 prevents passive ion diffusion through paracellular pathways, supporting gastric homeostasis and protecting epithelial cells from low pH conditions. Under physiological conditions, CLDN18.2 expression is strictly limited to the stomach (gastric) [19].

## 4. Claudin-18.2 Expression in GC

In epithelial-origin tumors, disturbances in the expression of genes encoding claudin family proteins are frequently observed. Overexpression is associated with alterations in the barrier (gate) function, often accompanied by a disorganized arrangement of tight junction strands, leading to increased permeability to paracellular markers. On the other hand, reduced claudin expression contributes to increased transcellular permeability of nutrients and growth factors into the tumor [8]. Lower claudin expression favors malignant transformation and epithelial–mesenchymal transition (EMT), which is associated with the loss of cell polarity, reduction in adhesive structures, and acquisition of migratory capabilities [19]. Thus, tight junctions become involved in the metastatic process of cancer.

During malignant transformation, ectopic expression of claudins is also observed, partly as a result of global hypomethylation in the cells, including the loss of methylation in the promoters of tissue-specific claudin genes. The loss of epithelial cell polarity during malignant transformation leads to the exposure of CLDN18.2 epitopes on the cell surface, which may have therapeutic significance [15,18].

CLDN18.2 is a highly selective biomarker whose aberrant expression is frequently observed during the development of primary malignancies such as gastric cancer (GC) and esophagogastric junction adenocarcinomas (EGJAs) [20]. The expression of CLDN18.2 has also been detected in other tumor types, including pancreatic, esophageal, biliary tract, ovarian cancers, head and neck cancer, bronchial cancer, and non-small-cell lung cancer [6,18,19]. Unlike in these tumors, in gastric cancer (GC), CLDN18.2 expression is typically reduced compared to the normal gastric mucosa. Even in precancerous conditions such as intestinal metaplasia or intraepithelial neoplasia, a decrease in CLDN18.2 mRNA levels is detected. In contrast, in precursor lesions of pancreatic cancer, an increased level of CLDN18.2 is observed [15]. Nevertheless, in gastric tumors, CLDN18.2 expression does not completely vanish—it remains relatively stable during the malignant transformation of the gastric epithelium and is detectable both in primary foci and in metastases (lymph nodes, liver) [16]. Immunohistochemical studies support this observation: while in normal gastric mucosa CLDN18.2 is highly expressed, its reduced expression has been found in approximately 58% of gastric cancer cases, and in tumors with an intestinal phenotype, up to 74%. In tumor cells, CLDN18.2 is expressed less frequently than in the surrounding mucosa or metaplastic tissue [18].

In one of the most comprehensive studies on this topic, CLDN18.2 expression was evaluated in 510 gastric cancer cases. High membranous expression of CLDN18 was found in 29.4% of primary tumors (150/510) and 34.1% of metastases, while aberrant localization (nuclear and/or cytoplasmic) was noted in 22.5% of primary tumors (115/510) and 25.0% (33/510) of metastatic cases. Membranous expression was more frequently associated with non-antral tumors, the diffuse type according to Lauren, and EBV-associated cancers [20].

In another study conducted between June 2012 and March 2016, immunohistochemistry was used to evaluate CLDN18.2 expression in a group of 430 patients with advanced gastrointestinal, genitourinary, or rare cancers. Expression was assessed in 414 of 430 patients (96.3%), and detected in 4.1% (17/414), including patients with pancreatic cancer (16.7%, 1/6), gastric cancer (14.1%, 12/85), biliary tract cancer (6.3%, 1/16), genitourinary/other cancers (2.2%, 1/46), and colorectal cancer (0.9%, 2/203). Of the 17 CLDN18.2-positive patients, 12 had gastric cancer (GC). In this subgroup, no statistically significant differences were noted in relation to gender, age, disease stage, primary tumor location, histological differentiation, HER2 status, or EBV infection. However, CLDN18.2 expression was significantly more frequent in the intestinal type compared to the diffuse type according to Lauren’s classification [19].

In pancreatic ductal adenocarcinoma, CLDN18.2 is expressed in 60–90% of cases. Among 5331 patients, 1585 (29.7%) showed CLDN18.2 expression. By cancer type, the prevalence was as follows: gastric cancer—39.3%, pancreatic cancer—54.5%, lung cancer—9.0%. Differences between studies stemmed from varying cut-off points, which did not use a clearly defined standard, and antibodies used in immunohistochemical assessments did not clearly specify whether the preparations used had the ability to distinguish CLDN18.1 from CLDN18.2. Furthermore, the studies had varying numbers of participants, with one study including almost ten times more patients than the other [21,22].

The frequency of CLDN18.2 expression in malignant tumors varies depending on the tumor type, study methodology, and scoring criteria adopted. In the context of metastasis, CLDN18 expression was lower in patients with liver and peritoneal metastases, but higher in those with lymph node and bone metastases. For example, in pancreatic cancers with ectopic CLDN18.2 expression, CLDN18.2 levels were higher in cases with lymph node metastases [18].

In a study conducted in a Japanese population, CLDN18.2 expression was found in 87% of primary tumors and 80% of lymph node metastases, with moderate-to-strong expression (≥2 + membrane staining intensity in ≥40% of cells) observed in 52% and 45% of cases, respectively. Notably, higher levels of CLDN18.2 expression correlated with the diffuse type of Lauren and tumors of higher malignancy grade [18].

### 4.1. CLDN18.2 Signaling Pathways

**Figure 2:**
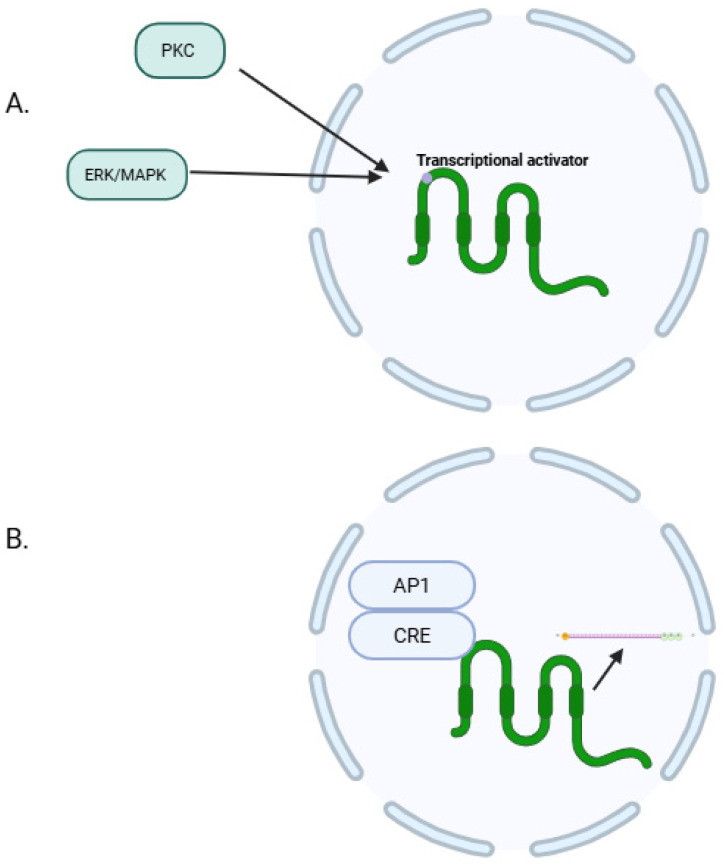
(**A**) PKC and ERK/MAPK pathways influence the regulation of CLDN18.2 expression through the modulation of an intracellular transcriptional activator. (**B**) AP-1 has the ability to bind to cis-regulatory elements (CREs) belonging to the CLDN18a2 promoter, thereby enhancing the transcriptional capability of CLDN18.2. Created in BioRender. Physiology, D. (2025) https://BioRender.com/6qk16yg.

CLDN18.2 plays an important role in various signaling pathways, starting with PMA (phorbol 12-myristate 13-acetate), which is primarily associated with the initiation of CLDN18.2 mRNA transcription, followed by translation. This occurs as a result of the phosphorylation of activated AP-1 and the activation of PKC (protein kinase C) and the ERK/MAPK (mitogen-activated protein kinase) pathway. PKC and ERK/MAPK pathways influence the regulation of CLDN18.2 expression through the modulation of an intracellular transcriptional activator (Figure 2A), while AP-1 has the ability to bind to cis-regulatory elements (CREs) belonging to the CLDN18a2 promoter, thereby enhancing the transcriptional capability of CLDN18.2 (Figure 2B) [23].

The application of inhibitors associated with PKC and MAP/ERK and ERKII leads to a decrease in the PMA-induced activity of the CLDN18.2 promoter [23,24]. The ERK/MAPK pathway in GC cells is responsible for regulating cell mobility. It is primarily involved in controlling MMP function in GCs, which contributes to the involvement of this pathway in cell migration and invasiveness [25]. In the case of the PKC pathway, its main role in GC is associated with the activation of JNK (c-jun N-terminal kinase), which in turn is associated with the induction of apoptosis within GC cells [26]. Moreover, CLDN18.2 activates multiple cellular signaling pathways associated with the Wnt β-linker effector. Among them are EFNB1, EFNB2, and CD44. In physiological conditions, this pathway influences the regulation of proliferation as well as the maintenance of stem cells. Moreover, it is associated with the maintenance of homeostasis in a properly functioning gastric mucosa. The Wnt/β-catenin signaling in GC is responsible for the development of this disease. Studies have shown a significant impact of this signaling in the self-renewal of GCSCs (gastric cancer stem cells) [27].

Additionally, it influences the HIPPO/YAP1 signaling pathways. The activation of this cascade leads to increased cell proliferation, as well as an enhancement of tumor cell growth, and also to the recurrence of the disease [28,29].

**Figure 3:**
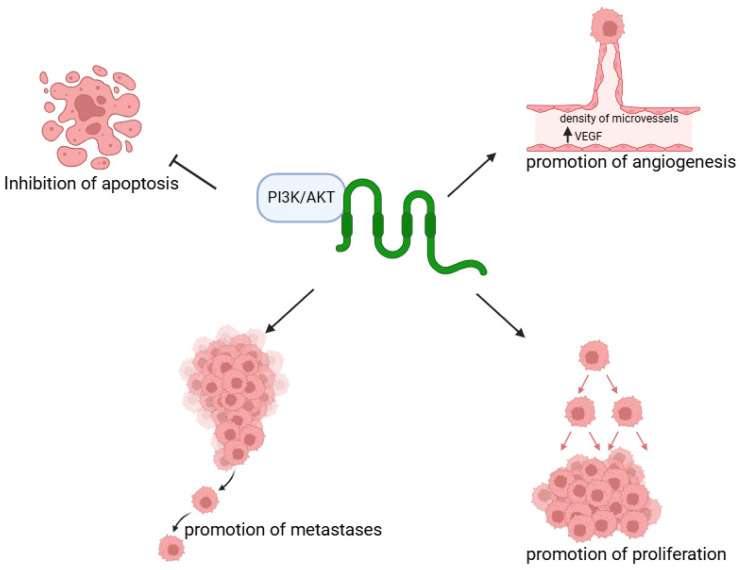
The role of the CLDN18.2-PI3K/AKT pathway on GC. Created in BioRender. Physiology, D. (2025) https://BioRender.com/1wp3c7j.

Furthermore, this claudin affects the activation of the PI3K/AKT pathway (Figure 3) [30]. In gastric cancer, this pathway is associated with anti-apoptotic activity, specifically integrated with the development of resistance in cancer cells to many factors related to the stimulation of apoptosis. The use of PI3K inhibitors negates this effect, consequently allowing for the execution of apoptosis. This is possible, among other things, due to the reduction in NF-κB (nuclear factor kappa-light-chain-enhancer of activated B cells) activity [31]. Moreover, the activity of the Akt is associated with the stimulation of processes aiming at the formation of metastases in stomach cancer [32]. p-Akt is positivity associated with the density of microvessels and VEGF, which affects angiogenesis among others in gastric adenocarcinoma [33]. On the other hand, dominant-negative Akt is responsible for inhibiting the proliferation of gastric cancer cells and also stimulates the blockade of the cell cycle at the G phase. Conversely, the upregulation of Akt positively influences the proliferation of cells [34].

It has been shown that HER2/HER is associated with maintaining appropriate barrier function by regulating the expression of CLDN18.2; however, the exact mechanism is not fully understood. Studies conducted to understand this signaling pathway have shown that the use of lapatinib, which is associated with inhibiting HER2, significantly affected IL-1β as an inhibitor of CLDN18.2 expression in the cell membrane. This indicates that IL-1β reduces the levels and distribution of CLDN18 in the cell membrane through the activation of the HER2/HER3 pathway, and the blockade of this pathway by lapatinib counteracts this effect [35]. HER2 in gastric cancer is responsible for invasion and the formation of metastases due to the stimulation of the RAF/ERK pathway [36]. HER2 overexpression correlates with greater aggressiveness of cancer, as well as with the possibility of tumor recurrence [37].

Moreover, miRNAs may also influence the regulation of CLDN18 expression, which is related to the ability of these molecules to bind to CLDN18 mRNA. Studies based on Western blot analysis and luciferase reporter assays have shown that miRNAs, and in particular, miR-1303, may contribute to the suppression of CLDN18.2 expression by binding to probable binding sites in the CLDN18 mRNA 3′-UTR [38]. miR-767, on the other hand, targets CLDN18.2, thus influencing the limitations related to the possibility of migration, proliferation, and invasion of cancer cells [39]. In general, miRNAs in GC influence proliferation, survival, migration, and invasion via the MAPK/ERK and PI3K/AKT signaling pathways [37].

There is evidence to support an inverse correlation between CLDN18.1 mRNA expression and promoter CpG island methylation. In vitro studies have shown that methylation of the CLDN18.1 gene promoter significantly inhibits the transcription of luciferase reporter constructs. These results suggest that CpG island methylation may limit the binding of transcription factors to the CLDN18.1 promoter, leading to reduced expression [40]. Moreover, the occurrence of CpG island methylation may cause absolute inhibition of the transcription factor CREB in binding to the CLDN18.2 promoter regions, which is necessary for the activation of its transcription [41].

### 4.2. GC Therapies Targeting CLDN18.2 Based on mAB

So far, many different but promising therapies targeting CLDN18.2 have been developed. One of them is the use of mABs—monoclonal antibodies designed to recognize and bind to CLDN18.2 within cancer cells. Their action aims to block the function of cancer cells or facilitate recognition by the immune system, leading to their destruction [42].

The most well-known and researched mAb against CLDN18.2 is Zolbetuximab (IMAB 362). It is a chimeric mouse antibody containing human IgG1 constant regions directed strictly against CLDN18.2 [18,43]. It is characterized by high affinity. There are no complications in the form of cross-reactivity with other types of claudins [18]. At the moment of binding to a specific epitope, its exposure occurs, which contributes to the activation of cytotoxicity mechanisms such as ADCC (antibody-dependent cellular cytotoxicity) and CDC (complement-dependent cytotoxicity), resulting in the apoptosis of cells in gastric cancer [43].

Many clinical trials have been conducted to evaluate the efficacy of Zolbetuximab in gastric cancer therapy (Table 1).

The phase II FAST study NCT01630083 investigated the addition of Zolbetuximab to first-line chemotherapy, in which patients were treated with EOX (epirubicin, oxaliplatin, and capecitabine). The expression threshold of CLDN18.2 was 40%. Patients received a dose of 600/800 mg/m^2^, maintaining favorable disease progression. Treatment demonstrated well-controlled adverse effects, resulting in high tolerance.

In the phase 3 SPOTLIGHT study NCT03504397, 215 patients were included who were administered Zolbetuximab at a dose of 800 mg/m^2^ in the first cycle. The dose was then reduced to 600 mg/m^2^. The expression threshold for CLDN18.2 was set at 75%. The use of this preparation significantly impacted the prolongation of progression-free survival. The most commonly observed adverse symptoms were primarily vomiting and nausea.

The phase III GLOW NCT03653507 double-blind study included 507 patients. The study arm included zolbetuximab in combination with CAPOX chemotherapy (capecitabine + oxaliplatin), and the control arm was CAPOX in combination with placebo. This combination significantly improved progression-free survival (PFS). However, it should be noted that there was no significant difference in the overall response rate (ORR) between the two groups. Adverse events reported with the treatment included nausea and vomiting. In addition, the NCT03505320 study will explore the possibility of using Zolbetuximab in combination with mFOLFOX6 along with Nivolumab.

Considering the results of both completed and ongoing studies, the FDA has prioritized zolbetuximab as a potential and promising first-line therapy for patients. The argument for this decision is primarily the limited information regarding the development of zolbetuximab resistance.

Another example of mABs is Osemitamab (TST001), which is also classified as a humanized antibody. It is an IgG1 characterized by better affinity in binding Fc to FcγRIIIa. In addition, it can trigger increased ADCC activity and exhibits a reduced toxicity profile. It can also interact with a very high efficacy rate on tumors characterized by moderate or even low expression of CLDN18.2 [28]. Moreover, the use of this antibody influences the upregulation of PD-L1 expression in cancer cells. Compared to the previously discussed Zolbetuximab, Osemitamab performed significantly better in inducing ADCC, CDC, and ADCP (antibody-dependent cellular phagocytosis) [44]. In the phase I/II study NCT 04495296, research is being conducted on a potential co-therapy with the anti-PD-1 antibody (nivolumab) in the treatment of gastric adenocarcinoma.

Additionally, a highly specific mAb for CLDN18.2 is Claudiximab (IMAB362), which is a chimeric IgG1 antibody derived from a monoclonal mouse antibody. To fulfill its role, Claudiximab underwent chimerization, allowing for the visualization of the constant region of human IgG1. In this case, there is also activation of ADCC and CDC. Furthermore, mAbs influence the activation of mechanisms related to apoptosis and affect the reduction in cell differentiation. In combination with chemotherapy, Claudiximab enhances T cell infiltration and stimulates inflammatory cytokines [45].

Another promising humanized mAB is ZL-1211. To enhance ADCC, ADCP, and CDC, mutations at the Fc region were introduced: S239D and I332E. This therapy does not have to be targeted only at tumor cells characterized by a high expression of CLDN18.2 but also at those that show moderate and low levels of CLDN18 expression. It is characterized by enhanced ADCC due to NK cell stimulation [46]. In the case of SPX-101, which is currently in the first phase of research—NCT05231733—it is directed at patients with solid tumors who have exhausted all possible treatment methods.

In the case of AB011, which is also a humanized IgG1 antibody targeting CLDN18.2, the preclinical study demonstrated effectiveness in the application of therapy with cytotoxic drugs [47].

The DR30303 is characterized by another structural approach. It is a humanized fusion protein containing an anti-CLDN18.2 heavy chain (VHH) Fc, which demonstrates considerable selectivity towards CLDN18.2. The use of VHH in mAb production is primarily associated with lower costs. This is mainly due to the ability of these antibodies to strongly bind to target antigens, allowing them to be created using an antibody library. Additionally, they can be produced using bacteria, which results in increased productivity, as a significant amount of antibodies can be obtained with a minimal financial outlay. This type of mAb also exhibits quite high stability, manifested by increased resistance to heat, denaturing agents, as well as surfactants [48]. Moreover, it is characterized by a significantly higher tumor penetration capability, as well as enhanced ADCC. This is made possible by the smaller size of DR30303 compared to other mAbs. The effectiveness of monotherapy has been tested on several mouse models obtained from xenografts of stomach and pancreas cells. The minimum dose that demonstrated therapeutic effectiveness was at a level of 0.3 mg/kg [48].

### 4.3. GC Therapies Targeting CLDN18.2 Based on bsAbs

A more promising approach seems to be the use of bsAbs (bi-spherical antibodies) in conjunction with ADCs (antibody–drug conjugates). A bsAb may consist of two monoclonal antibodies connected by a peptide linker. The formation of such a complex allows for binding to two different antigens or two distinct epitopes within the same antigen [18]. The use of bsAbs may guarantee precise stimulation of the immune response, which could additionally help to bypass the immune evasion mechanisms often used by cancers [25]. Most designed bsABs are associated with T cell activation (BiTE). Their biological basis of action involves immune targeting of tumor cells, which occurs through the collective integration of CD3 on T cells and a specific tumor antigen. Furthermore, other types of bsABs can be developed that are characterized by the ability to specifically target immune checkpoints, such as PD-1, as well as relevant cytokines and oncogenic signaling pathways [49].

One of these is the potential use of QLS31905 as a potential first-line treatment. QLS31905 is a bispecific antibody that targets CD3 and CLDN18.2. Its mechanism of action is related to the ability to target T lymphocytes within the tumor to induce cytotoxicity. Furthermore, this antibody is characterized by the release of low levels of cytokines, which translates into significantly fewer side effects associated with this therapy [50].

In the case of PT886, which, in addition to targeting CDLN18.2, also targets CD47, its primary function is to enhance the phagocytosis of tumor cells. This is achieved by inhibiting the CD47 signal, commonly referred to as the “don’t eat me” signal, which is often used by tumor cells to evade the immune system. This use of the antibody offers dual clinical advantages. It not only engages immune cells but also influences immune escape pathways [51].

Additionally, Q-1802 targets CLDN18.2 and PD-L1, which are associated with the potential to block immune checkpoints that closely impact tumor function. Early study results suggest promising antitumor activity in gastric adenocarcinoma, particularly in recurrent and refractory tumors [52].

The work of Yue et al. [53] describes a therapy using HC-2G4S (BsAb)—a bispecific antibody directed against two promising GC biomarkers, HER2 and CLDN18.2, which eliminates gastric cancer cells (expressing these two antigens) by enhancing the effector function of the immune system. This may prove to be an effective BsAb in the treatment of HER2-positive and/or CLDN18.2-positive gastric cancer, potentially overcoming resistance to, e.g., Trastuzumab.

The biggest drawback of using bsAB appears to be the occurrence of splicing variants, different isoforms of claudin family proteins, and the occurrence of various transmembrane structures. Furthermore, design issues may arise due to the limited extracellular regions of antigen exposure [21].

### 4.4. GC Therapies Targeting CLDN18.2 Based on CAR-T

A promising strategy appears to be immunotherapy based on chimeric antigen receptor (CAR)-modified T lymphocytes. It involves the use of genetic engineering to enhance the anticancer capabilities of T cells, which are isolated from the patient’s bloodstream. In the next step, they are modified through viral vectors. As a result, it is possible to introduce a chimeric antigen receptor [54]. The acquisition of this receptor enables an increase in the ability to recognize TAAs (tumor-associated antigens). Furthermore, these changes lead to the activation of T cell proliferation, increased cytotoxicity, and the transcription of genes that encode cytokines, thereby intensifying antitumor activity [55,56]. Subsequently, these modified cells are reintroduced into the patient’s bloodstream [54]. Within the CAR structure, three parts can be distinguished: a single-chain variable fragment (scFv), a hinge with a transmembrane domain, and an intracellular component. The scFv constitutes the extracellular fragment, which is primarily responsible for antigen recognition [54,57]. In its structure, scFv contains a sequence of light amino acids (VL) and a sequence of heavy amino acids (VH) of the antibody chains. Additionally, there is a short peptide that is connected to the hinge region, thereby affecting the ability to bind the antigen. In the case of the intracellular domain, its role is mainly associated with anchoring CAR-T within the cell membrane. Furthermore, it allows for signal transduction into the interior of the cell [58].

Data from preclinical studies with humanized autologous CAR-T targeting CLDN18.2 revealed high specificity for anticancer activity on GC and provided grounds for the use of this type of therapy [59]. In 2025, many studies on CAR T lymphocytes specific to CLDN18.2 were registered on clinicaltrials.gov (Table 2).

In the phase I clinical trial (NCT03159819), the main objective was to determine the safety, as well as the tolerance, and the cytokinetics of CAR-T transduced with a lentiviral vector targeting CLDN18.2. Additionally, preliminary efficacy of this therapy was sought. Patients received one or more cycles of CAR-CLDN18.2 T cell infusions. The therapy involved eleven individuals, of whom one achieved a complete remission of the disease. In other cases, no symptoms of severe toxicity or cytokine release syndrome were observed. It was also confirmed that CAR-T therapy is safe and well-tolerated by patients.

In the Phase I study designated as NCT03874897, CT041 was investigated, which is characterized by genetically and autologously modified lymphocytes possessing CAR expression directed against CLDN18.2. The study involved dose escalation of the preparation to assess the safety, immunogenicity, efficacy, and pharmacokinetics of CAR T therapy targeting CLDN18.2. The study included 28 patients with gastric cancer who were administered a dose of 2.5 × 10^8^ cells. In some cases, disease regression was observed. Adverse events occurred in the form of leukopenia, neutropenia, and anemia, but there were no symptoms of severe toxicity or cytokine release syndrome. NCT04404595 also includes a study on CT041. Five patients with gastric cancer were given a dose in the range of 2.5–4 × 10^8^ cells. In the case of one patient, CR (complete response) was achieved, and two patients had PR (partial response).

In NCT04977193, LY011 was included, which encompasses CAR T cell therapy of the third generation against anti-CLDN 18.2. It was produced from allogenic T lymphocytes modified to target the TAA within CLDN18.2. This is possible through the use of lentiviral vectors, which are characterized by potential immunostimulatory and anti-tumor effects. The main goal of the study is to assess efficacy and safety.

Another interesting therapy seems to be NCT05583201, which utilizes KD-496. It is a bispheric CART-T cell that not only has the ability to recognize CLDN18.2 but also NKG2D. In this way, the created CART-T cells can not only interact better with GC cells but also may eliminate the tumor more efficiently compared to single CAR-T cells. The study includes a possible dose increase. Additionally, safety, tolerance, and efficacy will be evaluated.

IMC002 is presented in the form of autologous cell therapy, which includes the VHH antibody that is characterized by quite significant specificity. There is no cross-reactivity. The study NCT05946226 aims to evaluate the possibility of applying a higher dose and thus to assess the safety and efficacy of the therapy.

CAR-T therapy is considered a breakthrough in cancer therapy, but it is associated with a number of significant challenges. Key difficulties include identifying highly tumor-specific targets and thoroughly understanding the complex properties of the solid tumor microenvironment (TME). These obstacles stem from the hostile nature of solid tumors, which create an environment unfavorable to the infiltration and effective functioning of T lymphocytes. This microenvironment is characterized by strong immunosuppressive properties, and cytokines play a crucial role in maintaining it. Advances in understanding the mechanisms of the immune response against cancer have created new opportunities for mechanistic studies aimed at overcoming these barriers. In this context, CLDN18.2 is emerging as a promising target for advanced cell therapies used in the treatment of solid tumors.

Unfortunately, targeted therapy in gastric cancer is often associated with the development of drug resistance. The main mechanisms of developing resistance to anticancer drugs are associated with the formation of new signaling pathways in cancer cells aimed at replacing those blocked by drugs. There are also often changes in the tumor microenvironment, its heterogeneity, and target proteins, which promote the development of resistance to targeted drugs.

## 5. Conclusions

Despite the enormous progress in research and discovery of modern therapies based on new molecular targets, there is still a lack of universal strategies for GC patients, hence the continuous search for new and promising therapeutic targets. Recently, claudin 18.2 (CLDN18.2) has joined this select group of very attractive candidates as a new biomarker for both diagnostic and therapeutic approaches in GC.

This biomarker is characterized by high expression during the formation of gastric tumors and also shows persistent expression in metastatic lesions; therefore, CLDN18.2 may be a suitable target in the treatment of gastric cancers. However, challenges remain, such as resistance mechanisms, the need for standardized diagnostic tests, and inter-patient variability in CLDN18.2 expression, with differences reaching up to 45% in various populations.

The results of the preliminary as well as defined stages of clinical trials with the involvement of mAB, bsAB, and CAR-T therapy indicate that these treatments are characterized by a fairly high safety profile and show promising results related to clinical efficacy. Therefore, it is very important to unveil the results of ongoing clinical trials to gain even more data.

However, challenges remain, including interpatient CLDN18.2 expression variability, as well as an appropriate definition of the population associated with high expression of CLDN18.2, which could significantly benefit the use of treatment. Furthermore, this will allow the determination of the likelihood of effectiveness of the applied marker and the implementation of appropriate safety management systems in the context of adverse events. Another action should include an appropriate treatment regimen encompassing combination therapies (e.g., with immune checkpoint or HER2 inhibitors) and expanding applications to other CLDN18.2-positive tumor types.

In summary, CLDN18.2 represents a novel, tumor-specific biomarker with significant potential for future use in oncology therapies, especially since it may act as an oncogene or a tumor suppressor gene.

## Figures and Tables

**Table 1:** Registered studies on Zolbetuximab (mAB specific for CLDN18.2) in GC therapy.

Trial Number	Conditions	Status/Phase	Age	Locations
NCT06962137	Gastric cancer adenocarcinoma metastatic	Not yet recruiting	≥18	Belgium
NCT06881017	Gastric cancer	Not yet recruiting	≥18	-
NCT01630083	Gastric cancer adenocarcinoma	Completed	≥18	Bulgaria, Czechia, Germany, Latvia, Russian Federation, Ukraine,
NCT01671774	Gastric cancer adenocarcinoma	Completed	≥18	Germany, Latvia
NCT06048081	Locally advanced unresectable gastric cancer adenocarcinoma; metastatic gastric adenocarcinoma cancer	Available	≥18	USA, Brazil, France, Germany, Republic of Korea, Singapore
NCT03505320	Gastric cancer	Active, not recruiting	≥18	USA, France, Italy, Republic of Korea, Taiwan
NCT03653507	Locally advanced unresectable gastric cancer adenocarcinoma; metastatic gastric adenocarcinoma cancer	Active, not recruiting	≥18	USA, Argentina, Canada, China, Croatia, Greece, Ireland, Japan, Republic of Korea, Malaysia, Netherlands, Portugal, Romania, Spain, Taiwan, Thailand, Turkey, UK
NCT03504397	Locally advanced unresectable gastric cancer adenocarcinoma; metastatic gastric adenocarcinoma cancer	Active, not recruiting	≥18	USA, Belgium, Brazil, Canada, Chile, China, Colombia, France, Germany, Israel, Italy, Japan, Republic of Korea, Mexico, Peru, Poland, Spain, Taiwan, UK
NCT06901531	Locally advanced unresectable gastric cancer adenocarcinoma; metastatic gastric adenocarcinoma cancer	Not yet recruiting	≥18	-

**Table 2:** Registered studies on CAR T lymphocytes specific to CLDN18.2 in GC therapy.

Trial Number	Conditions	Status/Phase	Cells	Age (Years)	Locations
NCT03159819	Gastric adenocarcinoma	Unknown	-	18–70	China
NCT03874897	Advanced gastric cancer	Completed	CT041	18–75	China
NCT05472857	Gastric cancer	Recruiting	IMC002	18–70	China
NCT04404595	Gastric cancer	Active, not recruiting	CT041	18–76	USA and Canada
NCT04977193	Advanced gastric adenocarcinoma	Unknown	LY011	18–70	China
NCT05583201	Gastric cancer	Recruiting	KD-496	18–75	China
NCT05393986	Gastric adenocarcinoma	Unknown	CT048	18–75	China
NCT05620732	Gastric cancer	Recruiting	-	18–75	China
NCT04581473	Gastric adenocarcinoma	Recruiting	CT041	18–75	China
NCT06353152	Gastric adenocarcinoma	Recruiting	-	18–70	China
NCT05952375	Gastric cancer	Recruiting	XKDCT086	18–75	China
NCT05539430	Gastric cancer	Recruiting	LB1908	18–75	USA
NCT05946226	Gastric cancer	Recruiting	IMC002	18–70	China

## Data Availability

Not applicable.

## Abbreviations

The following abbreviations are used in this manuscript:

ADCC	Antibody-dependent cellular cytotoxicity
ADCP	Antibody-dependent cellular phagocytosis
BsAb	Bispecific antibody
CAR	Chimeric antigen receptor
CDC	Complement-dependent cytotoxicity
CEA	Carcinoembryonic antigen
CLDN18.2	Claudin 18.2
CR	Complete response
CREB	cAMP response element-binding protein
EGJA	Esophagogastric junction adenocarcinomas
GC	Gastric cancer
HER2	Human epidermal growth factor receptor 2
ICPM	Immune checkpoint molecules
mAB	Monoclonal antibody
MAPK	Mitogen-activated protein kinase
PKC	Protein kinase C
PR	Partial response
scFv	Single-chain variable fragment
TJ	Tight junction
